# Heat and solvent resistant electrospun nanofibers for tunable fluid transport in lateral flow applications

**DOI:** 10.1038/s41598-025-31691-x

**Published:** 2026-01-14

**Authors:** Nathan K. Bentley, Johan Liotier, Hanyan Lyu, Theresa Peters, Thomas Brandstetter, Jürgen Rühe

**Affiliations:** 1 Dept. of Microsystems Engineering (IMTEK), University of Freiburg, IMTEK, Georges-Köhler-Allee 103, 79110 Freiburg, Germany; 2 livMatS@FIT, University of Freiburg, Georges-Köhler-Allee 105, 79110 Freiburg, Germany; 3 FIT – Freiburg Institute of Interactive Materials and Bioinspired Technologies, University of Freiburg, Georges-Köhler-Allee 105, 79110 Freiburg, Germany

**Keywords:** Chemistry, Materials science

## Abstract

Lateral flow devices (LFDs) rely on porous materials to guide liquid transport, and electrospun fiber mats have emerged as attractive candidates due to their high surface area, tunable porosity, and ease of functionalization. However, conventional electrospun nanofibers often deform or dissolve when exposed to heat or to solvents commonly used in biochemical assays, limiting their applicability. To address this challenge, we developed electrospun poly(methyl methacrylate) (PMMA) nanofibers that are both heat-stable and solvent-resistant through the incorporation of photoreactive groups and subsequent solid-state crosslinking via the C, H-insertion crosslinking (CHic) reaction. PMMA fibers containing methacryloyloxy benzophenone (MABP) units were rapidly activated with UV light, yielding nanofiber mats that retain their morphology under elevated temperatures and also in solvents typically used in biochemical assays. Beyond mechanical and chemical stability, we demonstrate that the surface energy of these fibers can be precisely tuned through the formation of a crosslinked hydrogel sheath composed of dimethyl acrylamide (DMAA) and MABP comonomers. This sheath can be applied either by dip-coating of mats of pre-formed fibers or by coaxial electrospinning, and becomes covalently integrated with the fiber core upon light activation. The resulting core–sheath structure transforms the originally hydrophobic PMMA mat into a highly hydrophilic, paper-like material capable of supporting controlled capillary flow. These robust and tunable nanofiber mats offer new opportunities for the design of lateral flow devices with improved solvent compatibility, thermal stability, and flow performance.

## Introduction

Lateral flow devices (LFDs), often in the form of test strips, are simple devices that analyze a liquid sample containing the analyte of interest^1^. A defining feature of these devices is that the liquid sample moves along the strip through capillary action and therefore, pumps are not needed for liquid transport^2^. The pad or test strip is made of paper or, more frequently, nitrocellulose. The presence of target molecules is determined by the interaction of immobilized probe molecules on the strip’s surface in the form of a test line^3^. A second line, the control line, is typically printed a little further downstream on the test strip, to ensure that the test is fully functional. This line should always give a positive signal as long as the test strip is fully functional. The probe molecules interact with the analyte of interest, which is accompanied by a color change. These binding events can be detected through colorimetric or fluorescence readout. LFDs are widely used in medical diagnostics in various scenarios, such as at-home tests (e.g., pregnancy or SARS-CoV-2 tests), point-of-care tests (e.g., Troponin tests), and laboratory tests^3^.

When the analyte concentration is very low, a simple colorimetric readout is no longer suitable because the sensitivity becomes too low. This is even the case when complex, strongly light-absorbing chromophores, such as gold nanoparticles, are used for signal enhancement. Fluorescence analysis is commonly used in molecular diagnostics to address this issue^4^. However, the autofluorescence of nitrocellulose, the most common material used for LFDs, is rather high. This high background makes read-out of low-concentration analytes difficult, if not impossible. In contrast to this most polymeric materials without special chromophores tend to have lower autofluorescence than nitrocellulose^5^. They must also mimic the highly porous structure of paper-like materials. One way to achieve this and obtain high surface-area fibers would be to generate mats consisting of electrospun fibers.

The concept of electrospinning as we know it today was conceived in 1887 by Charles V. Boys. Boys was the first to produce fibers from a viscoelastic liquid via an external electric field^6^. Over the next century, the electrospinning technique was refined and developed significantly. Its uses span a large number of applications, ranging from air filtration to catalysis, and materials ranging from organic polymers to composites^7,8^. Electrospinning can produce fiber diameters ranging from several micrometers to tens of nanometers. Some reports even report fiber diameters of less than 1 nm^9,10^. The basis of electrospinning is the formation of a jet through the application of a high potential to a liquid droplet at the tip of a spinneret. This results in the stretching and elongation of the jet and the production of fibers^11,12^. Advancements in the process, including fiber alignment, coaxial electrospinning, and large-scale production using both multiple-needle and needleless electrospinning, have significantly enhanced the potential of this technique across various applications and research areas^13^.

Like the bulk polymer, the fibers produced via electrospinning are naturally susceptible to thermal deformation and dissolution. Therefore, exposure to heat or solvents during processing or use can deform or destroy the fibers, which leads to fiber degradation and negatively affects their use in certain applications. To avoid such stability issues in bulk systems, many physical and chemical crosslinking techniques have been developed, designed to form stable networks from appropriate precursor polymers^14^. Crosslinking improves properties such as mechanical strength, solvent and thermal stability, and, in some cases, biological compatibility^15^. Although the addition of a low-molecular-weight crosslinker during fiber generation allows straightforward generation of crosslinked networks in polymer-analogous reactions, this process cannot be used in typical electrospinning processes. One of the reasons is that residual crosslinker molecules (and perhaps catalysts), which are quite highly reactive, could leach out of the fibers during use and lead to problematic situations. Furthermore this process requires intrinsically the use of polymers containing appropriate reactive groups^16^.

An attractive approach to generating crosslinked polymer networks is the C, H-insertion crosslinking (CHic) reaction. To obtain such coatings first a layer of polymer is deposited using physical deposition techniques such as dip or spray coating. The CHic process is based on the formation of reactive intermediates which are generated when the dormant crosslinker units are activated. During activation, which can be photochemical or thermal, reactive moieties are formed, including ketyl biradicals, carbenes and nitrenes, depending on the type of crosslinker. For instance, ketones form upon irradiaton an excited biradicaloid triplet state. These biradicals can react with nearby C-H groups, forming covalent bonds between two^15,17–21^. The CHic process enables crosslinking of polymers in the solid state and simultaneous attachment to organic substrates or inorganic substrates covered with a suitable primer.

This study examines the copolymerization of methacryloyloxy benzophenone (MABP) and methyl methacrylate (MMA) to form the copolymer PMMA-co-MABP. MABP is a monomer containing a crosslinker moiety that can be photochemically activated. The resulting copolymer was then subsequently used in the electrospinning process. To modify the surfaces of the fibers, they are coated with a sheath made of a second, also CHic-able copolymer. This sheath layer can be applied directly during fiber formation via coaxial electrospinning or by dip coating of the fiber mats using a solution of the sheath material. For instance, the coating can be composed of a hydrogel precursor polymer made of dimethylacrylamide (DMAA) copolymerized with MABP. During photochemical activation both the core and the sheath of the fibers become crosslinked. At the same time, the sheath becomes covalently attached to the core. Through this core-sheath strategy, the surface properties of electrospun PMMA copolymers can be altered in a tailored fashion. This study examines the thermal and solvent stability of the fiber mats, their water imbibition behavior, and their surface properties. Finally, a first simple application of the obtained material in the form of an LFD is described.

## Results

**Fig. 1:**
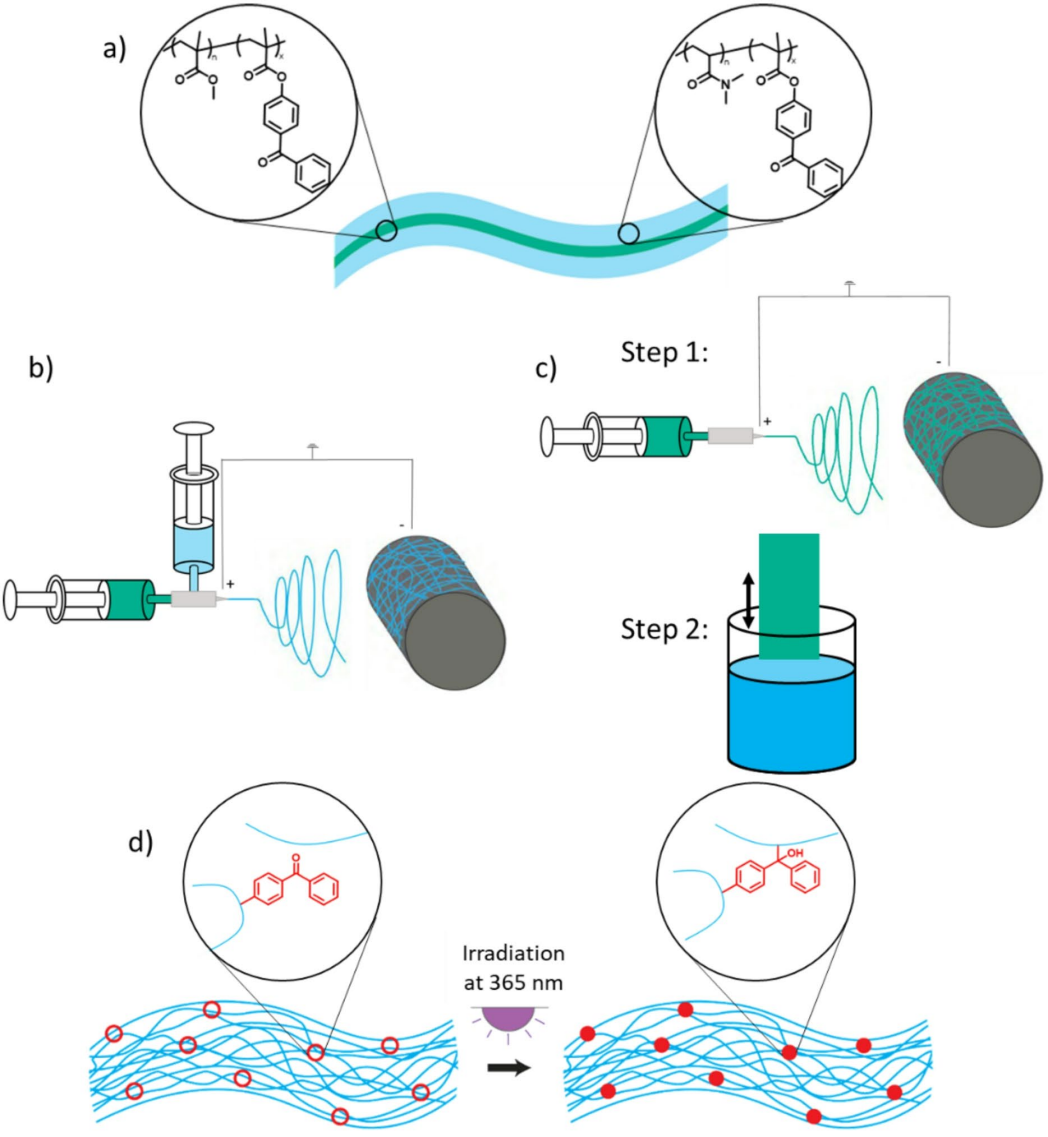
(a) Schematic depiction of the generation core-sheath structures by electrospinning. (b) Functionalized fiber mats are obtained in a one step using coaxial electrospinning or (c) in two steps by electrospinning of the core material followed by dip coating of the sheaf material. (d) Subsequent to fiber formation, the polymers forming the fibers are then crosslinked by irradiation at a wavelength of λ = 365 nm.

To achieve flexible, resistant fibers with good wetting properties, we created a core-sheath structure (Fig. 1a). The core provides the necessary flexibility and stability. It is made of a standard PMMA matrix polymer that contains small amounts of the photocrosslinker MABP. The sheath is made of a copolymer consisting of a hydrophilic PDMAA matrix, as well as MABP, which controls the surface properties, especially the wetting properties. For an LFD usage, the fibers must be hydrophilic, and the sheath and the core must be stable under operating conditions. To ensure this, we incorporated MABP crosslinker units into the polymers which can crosslink the material upon brief UV-irradiation, enhancing its stability^22^.

There are two ways to obtain core-sheath structures. In the first approach coaxial electrospinning of both polymers is performed. This method produces the core-sheath structure in one step (Fig. 1b). The second option is simpler, but it requires two steps (Fig. 1c). First, the PMMA core is electrospun alone, and then the resulting mat is dip-coated in a PDMAA copolymer solution to generate the sheath.

Crosslinking of the core and sheath is based on the well-established C, H insertion crosslinking (CHic) methodology and occurs during UV-irradiation (Fig. 1d). We decided to irradiate at a wavelength of 365 nm to enhance the penetration depth of the light and ensure the crosslinking of both the sheath and the core of the fibers. Additionally, the CHic reaction covalently links to the nearest C-H bond once activated, regardless of the polymer to which it belongs. This covalent bonding between the core and sheath materials prevents delamination.

### Electrospinning of CHic copolymers

**Fig. 2:**
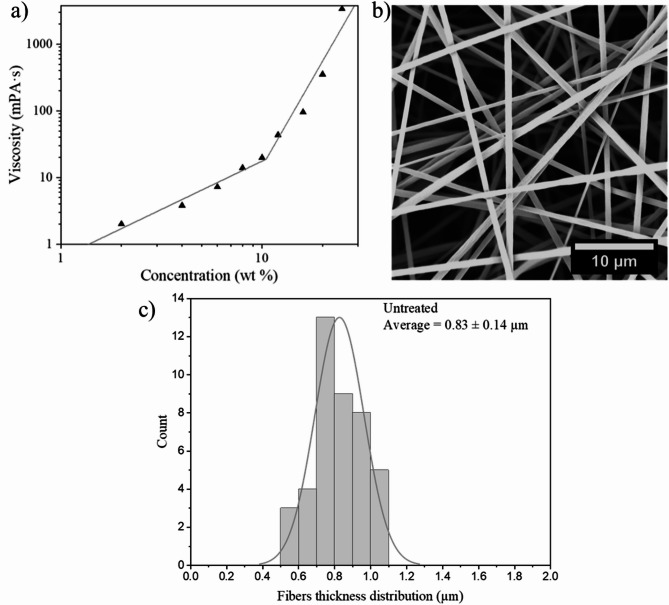
(a) Chart of concentration vs. viscosity of PMMA-15%MABP (M_w_ = 230 kDa) in DMF plotted on a log-log scale. (b) SEM image of PMMA-15%MABP fibers electrospun from DMF (c = 20 wt%, T = 30 °C, Rh = 20%, V = + 10.5 kV, -3 kV, flow rate = 50 µl·min^-1^) after UV-crosslinking at λ = 365 nm (dose: 10 J·cm^-2^). (c) Size distribution of the untreated fiber mats obtained from PMMA-15%MABP.

As far as the polymer is concerned, one of the most important parameters for obtaining defect-free fibers by electrospinning is the viscosity of the polymer solution. Accordingly, the concentration and molecular weight of the polymer chains, as well as the quality of the solvent are important parameters. In this study, the polymer’s molecular weight of was either 160–230 kg/mol, depending on the copolymer composition, while the solvent remained constant. Thus, prior to electrospinning, it is important to determine the entanglement concentration to understand the minimum and optimal concentrations for producing defect free fibers. The optimal concentration is generally assumed to be approximately 2-2.5 times larger than the entanglement concentration^23^ which can be determined by measuring the viscosity at various concentrations and plotting the data on a log-log scale. Figure 2a shows the resulting curve obtained using PMMA-15%MABP in DMF. The figure clearly shows an inflection point at approximately 10 wt%, which is the entanglement concentration of the polymer solution. Therefore, the ideal electrospinning concentrations is assumed to be between 20 and 30 wt%. Figure 2b shows a scanning electron microscope (SEM) micrograph of PMMA-15%MABP electrospun at this concentration. The figure shows well-formed, defect-free fibers, with thicknesses of 830 nm ± 140 (Fig. 2c). The same experiments were conducted with P(DMAA-5%MABP), which also gave well-formed fibers (Fig. 9).

### Heat and solvent resistance of crosslinked fiber Mats

**Fig. 3:**
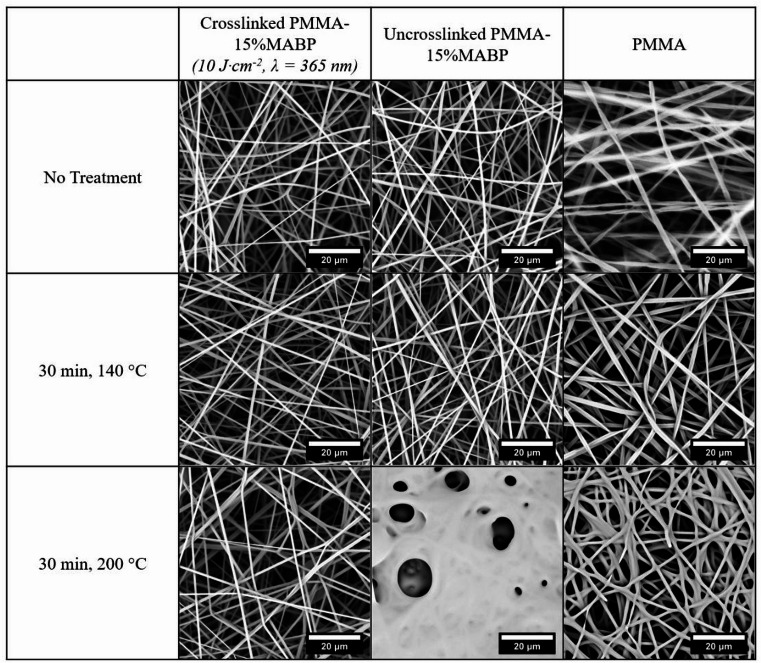
SEM micrographs of electrospun nanofiber mats after heating in the oven for 30 min at 140 °C and 200 °C. Nanofibers containing crosslinker units (activated and unactivated) and nanofibers without crosslinker units are compared. The PMMA-15%MABP fibers were electrospun from DMF (conc. = 20 wt%, T = 30 °C, Rh = 20%, V = + 10.5 kV & -3 kV, flow rate = 50 µl·min^-1^, Mw = 230 kDa). The PMMA (350 kDa) fibers were electrospun from DMF (T = 30 °C, Rh = 20%, V = + 12 kV & -3 kV, flow rate = 20 µl·min^-1^).

To investigate the effect of crosslinking on the thermal stability of the fibers, fiber mats of PMMA-15%MABP and PMMA without a crosslinker were electrospun from DMF. Some samples from the PMMA-15%MABP mat were irradiated with UV-light (365 nm, 10 J·cm^− 2^), while others were not. We checked the thermal stability of the electrospun fiber mats by heating the samples for 30 min at two different temperatures above the polymer’s T_g_ (~ 105 °C), with one below and one above the melt flow temperature (~ 160 °C). The resulting SEM micrographs taken after heating can be seen in Fig. 3. Minimal changes were observed at 140 °C, though slight thermal deformation was noted in the non-crosslinked PMMA-15%MABP and the pure PMMA fibers. However, after heating to 200 °C, significant damage was observed in all non-crosslinked fibers, to the point that they completely clumped together and melted. The crosslinked fibers in contrast demonstrate good stability even after being heated at 200 °C for 18 h (see Fig. 10).

**Fig. 4:**
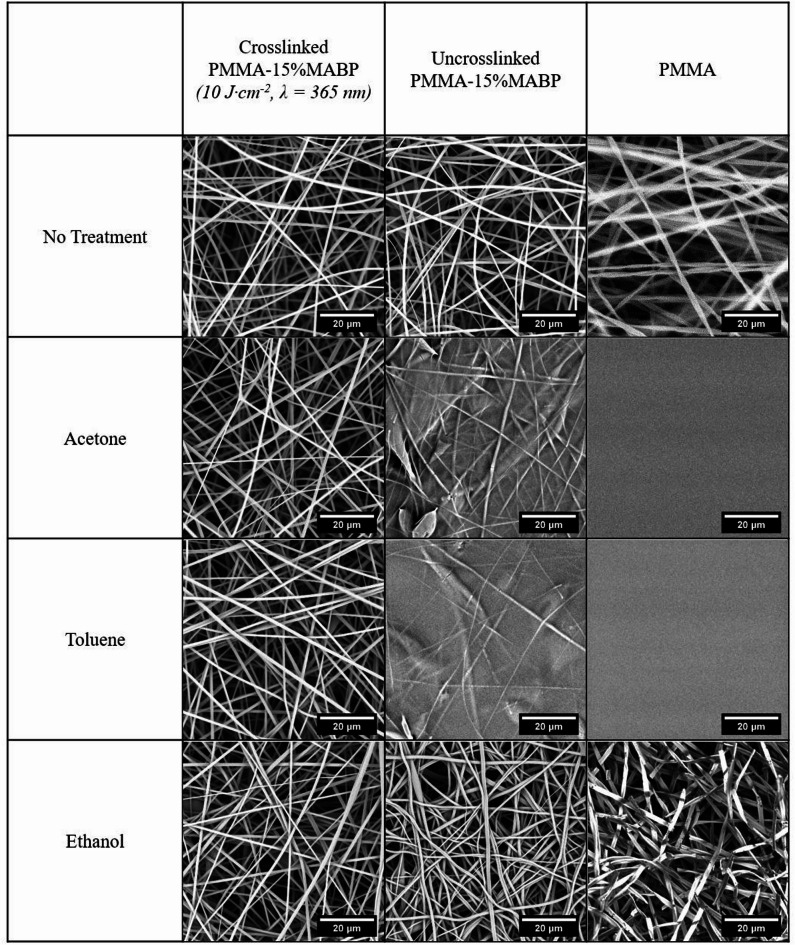
SEM micrographs of electrospun nanofiber mats after the addition of various solvents. Nanofibers containing crosslinker units (activated and non-activated) and nanofibers without crosslinker units are compared. The PMMA-15%MABP fibers were electrospun from DMF (conc. = 20 wt%, T = 30 °C, Rh = 20%, V = + 10.5 kV & -3 kV, flow rate = 50 µl·min^− 1^, Mw = 230 kDa). The PMMA (350 kDa) fibers were electrospun from DMF (T = 30 °C, Rh = 20%, V = + 12 kV & -3 kV, flow rate = 20 µl·min^− 1^).

Similar methods were used to examine the solvent resistance of the fiber mats. Fiber mats with and without crosslinker were electrospun and then exposed to various solvents. As seen in Fig. 4, the crosslinked PMMA-15%MABP fibers were unaffected by any of the tested solvents. These fibers were then immersed in toluene for 18 h, and there were no signs of degradation (see Fig. 11). A slight deformation was observed, which is attributable to swelling during the extended exposure. In contrast, the non-crosslinked PMMA-15%MABP and PMMA mats significantly deformed when exposed to acetone and toluene. The PMMA fibers dissolved completely after exposure to both solvents, forming a thin film. The non-crosslinked PMMA-15%MABP mats retained some fiber structure but essentially transformed into a thin film, with no visible pores between fibers. It is expected that acetone and toluene, which are both good solvents for these polymers, would strongly affect the non-crosslinked fibers. However, the impact of ethanol is more surprising. Despite being a poor solvent for these polymers, ethanol still caused partial dissolution and deformation, highlighting the vulnerability of the non-crosslinked fibers, which are prone to damage even from weak solvents.

### Alteration of wetting properties through hydrogel sheaths

**Fig. 5:**
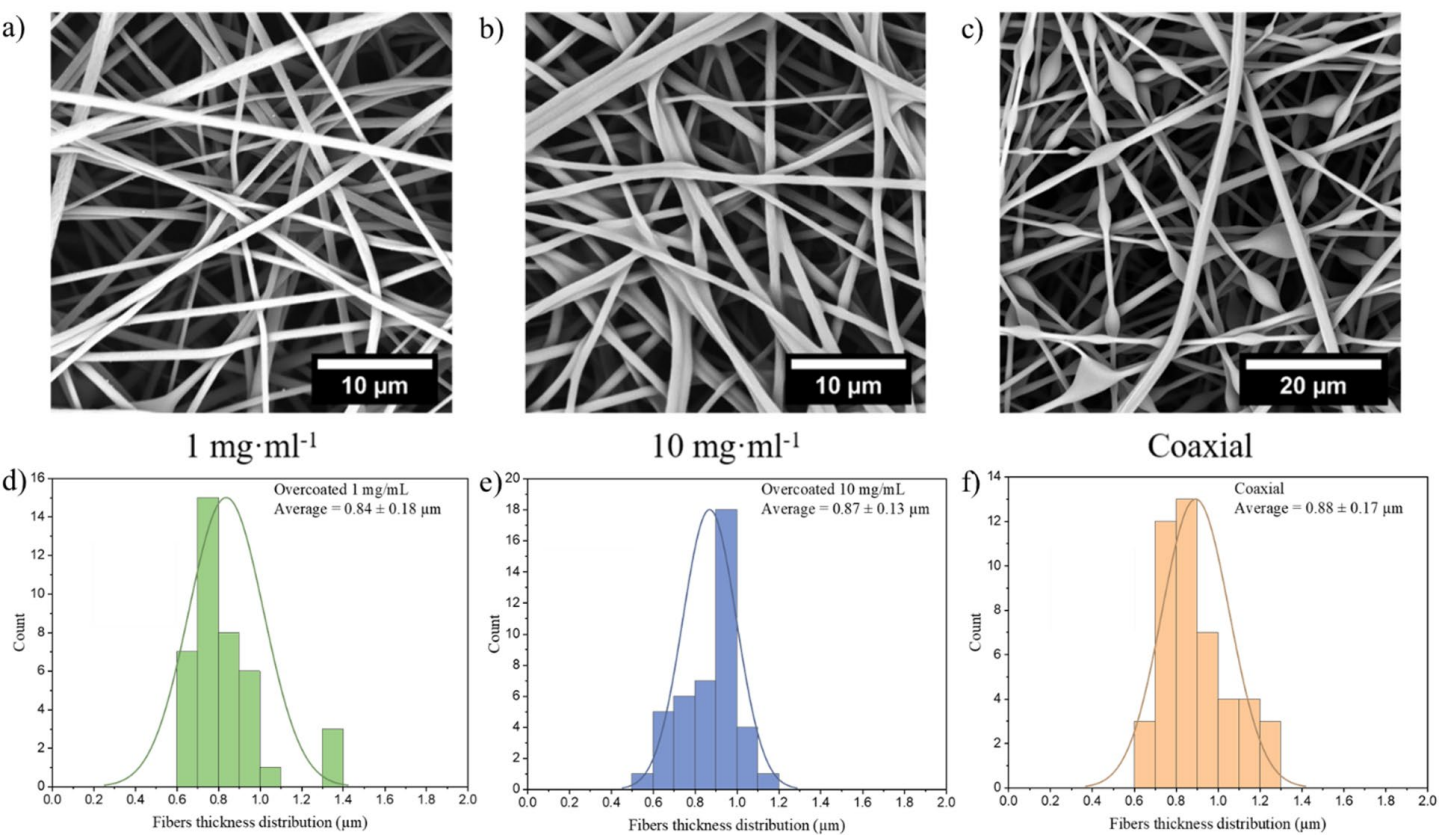
SEM micrographs of PMMA-15%MABP fibers electrospun from DMF (c = 20 wt%, V = + 10.5 kV, -3 kV, flow rate = 50 µl min^-1^), crosslinked (10 J cm^-2^ at 365 nm), and dip coated with PDMAA-5%MABP at concentrations of (a) 1 mg ml^-1^ and (b) 10 mg ml^-1^ and their respective size distribution (respectively d and e). (c) SEM micrograph of a nanofiber mat produced via coaxial electrospinning with a PMMA-15%MABP (Mw = 230 kDa) core and a PDMAA-5%MABP (Mw = 95 kDa) sheath. The solutions were electrospun from concentrations of 21 wt% and 30 wt% from DMF, respectively using the same conditions (V = + 10.5 kV, -3 kV, flow rate = 50 µl min^-1^) and crosslinked with a dose of 10 J cm^-2^ at 365 nm and its size distribution (f).

The wetting properties of the fiber network can also be adjusted by generating a fiber with a hydrophilic surface. This can be achieved by using pure hydrogel fibers made of PDMAA-co-MABP that are crosslinked with UV-light (365 nm, 10 J·cm^− 2^). However, upon water exposure, the hydrogel’s strong swelling nature results in permanent deformation of the fibers and the fiber mats. Therefore, PMMA-15%MABP fibers were crosslinked (365 nm, 10 J cm^− 2^) and dipped a PDMAA-5%MABP solution, followed by a second crosslinking step (365 nm, 10 J·cm^− 2^). This created a core-sheath fiber structure in which the sheath was covalently linked to the fiber surface and did not come off, even after prolonged extraction with a solvent that dissolves the sheath polymer. SEM micrographs of the fibers coated with polymer solutions at two different concentrations (1 mg ml^− 1^ and 10 mg ml^− 1^) can be found in Fig. 5a and b. The core-sheath fibers can also be produced simultaneously using coaxial electrospinning. A SEM micrograph of these fibers is shown in Fig. 5c.

**Fig. 6:**
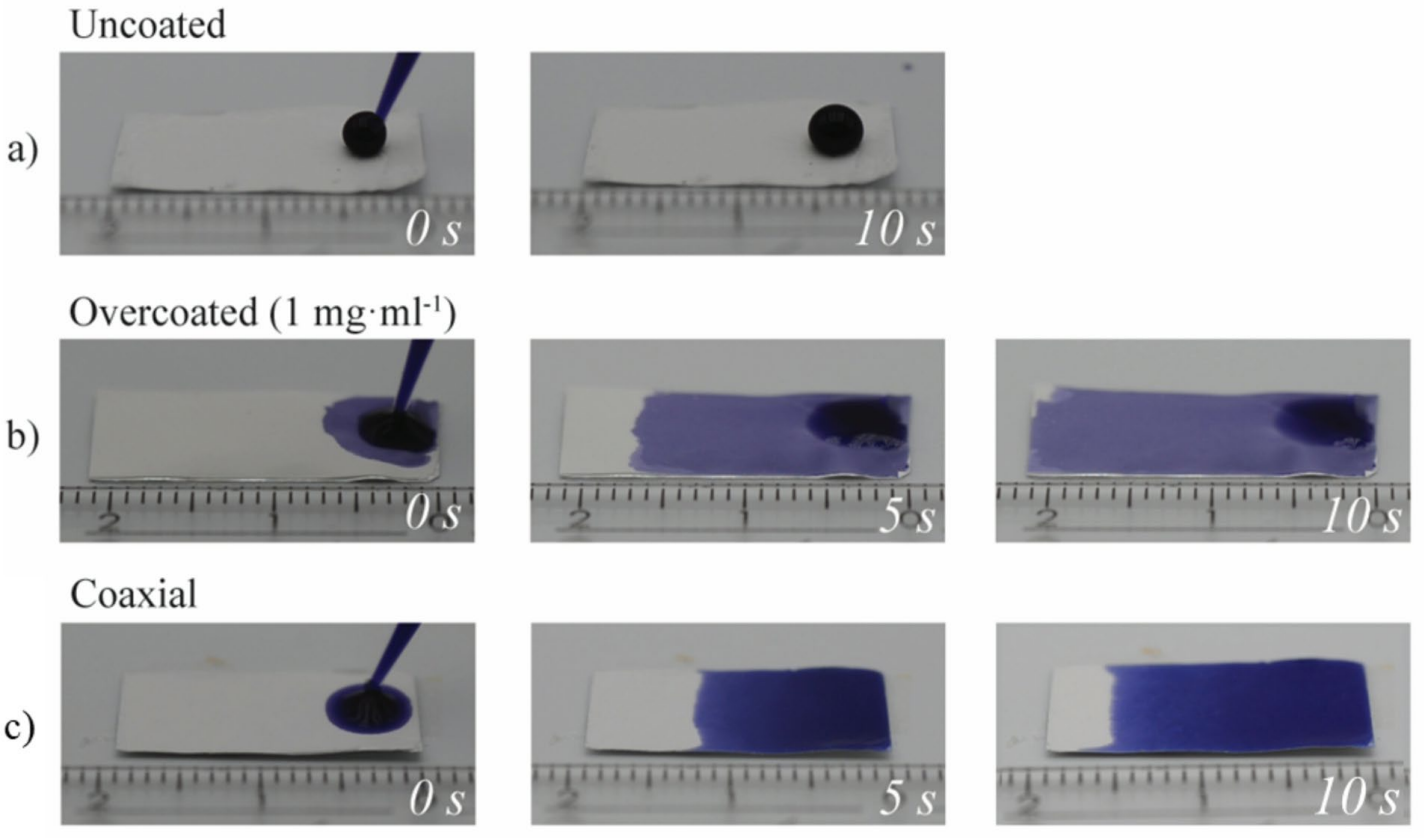
Flow of a 40 µl water droplet pipetted onto (a) uncoated PMMA-15%MABP fibers, (b) coated PMMA-15%MABP fibers (dip coated in 1 mg·ml^− 1^ PDMAA-5%MABP in ethanol, 50 mm·min^− 1^, 10 s immersion), (c) coaxial electrospun fibers (PMMA-15%MABP core, PDMAA-5%MABP sheath) after various times (indicated on image).

To demonstrate the improved flow properties of the core-sheath fiber structure, a 40 µl droplet of water containing a blue dye was pipetted onto strips of fiber mats.The resulting flow of the liquid was then observed. Images showing the progression of the imbibition process can be seen in Fig. 6. When the unmodified mat was used, the water droplet did not wet the surface at all, forming an almost perfect droplet. Strong flow was observed on both mats consisting of the core-sheath fibers, i.e., on the mats that had been dip-coated (b) and on mats obtained by coaxial spinning (c).

**Fig. 7:**
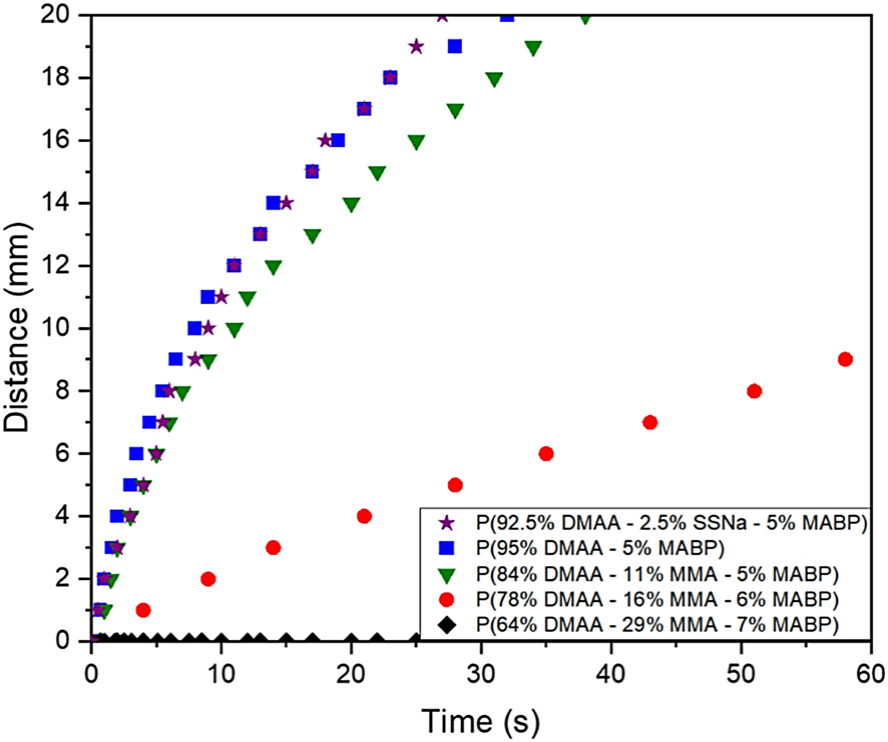
Flow of a 40 µL droplet placed on electrospin PMMA fiber mats (0.5 cm x 2.5 cm) coated by copolymers with different hydrophilicity as a function of time.

Introducing ionic or hydrophobic moieties into the polymer allows fine-tuning of its imbibition properties. Figure 7 shows the flow of a water droplet on fiber mats coated with copolymers of varying hydrophilicity. To enhance the hydrophilicity of PDMAA, we incorporated an ionic unit, sodium styrene sulfonate (SSNa). The resulting polymer exhibits a flow behavior similar to that of pure PDMAA, but the droplet reaches the 2 cm mark slightly faster (27 s for the ionic copolymer versus 32 s for PDMAA).

In contrast, incorporating hydrophobic MMA units at different contents (11%, 16%, and 29%) produces a markedly different response. The copolymers containing 11% and 16% MMA show significantly slower flow, with the droplet reaching 2 cm after 38 s and 166 s, respectively. For the copolymer with 29% MMA, no flow was observed.

### Application of electrospun mats to LFDs

**Fig. 8:**
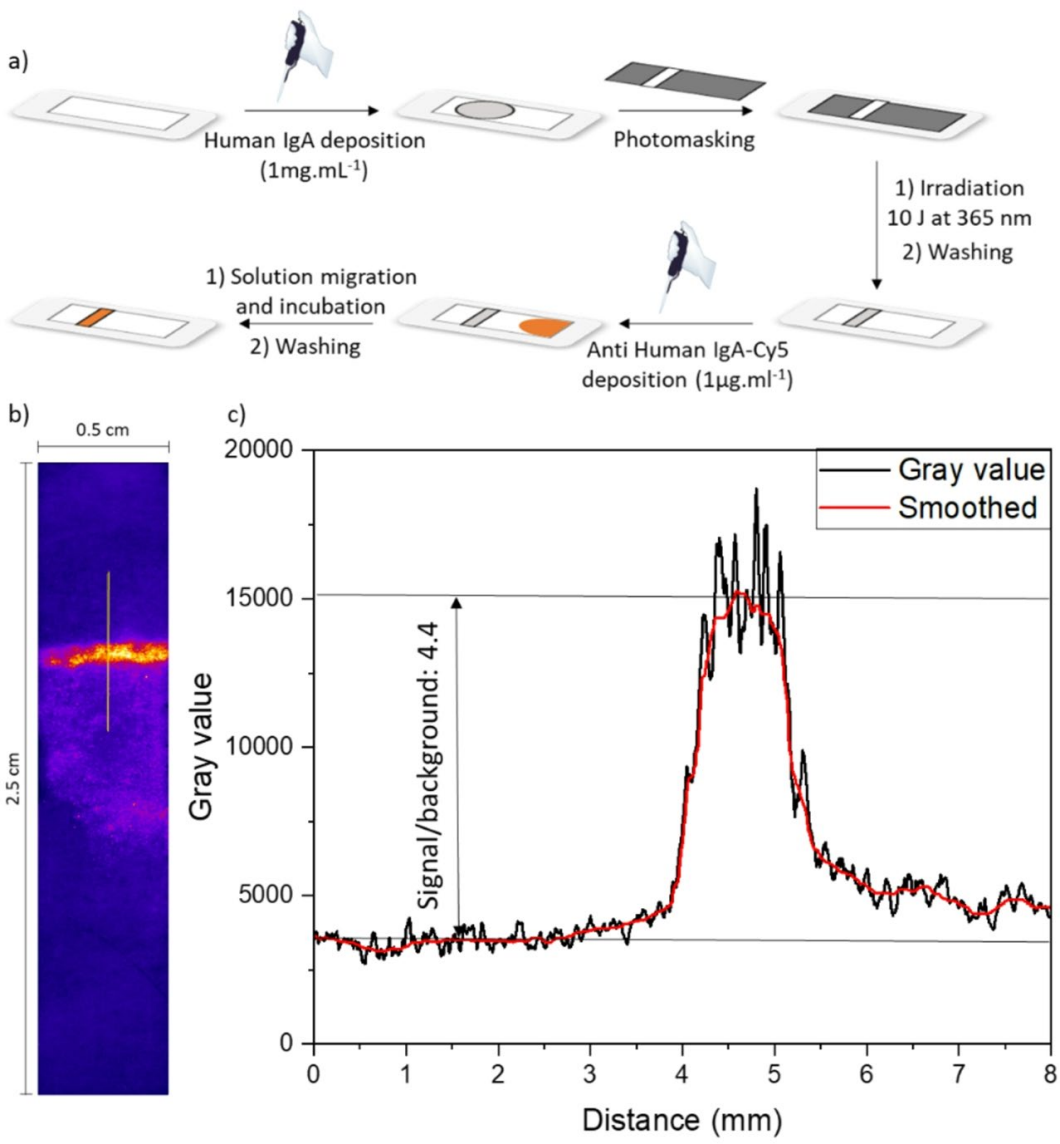
(a) Schematic depiction of the process of lithographically defining the detection line in the assay (b). After incubation of the test strip with the analyte solution, the sample was analyzed in a fluorescence reader yielding the image (b); sample size 2.5 cm/0.5 cm) (c) gray value profile from which the grey value is extracted (the yellow line in (b) shows the where the profile was taken).

Printing the detection line on the LFD is a critical yet challenging task. The LFD requires a hydrophilic material in order to achieve a fast water imbibition and analyte transport along the strip. However, this also makes the printing of the line difficult because the components containing the probes are also water-soluble and spread during deposition, resulting in a frayed and blurry detection line. To obtain a sharp line, we used a PMMA-MABP mat coated with PDMAA-MABP and crosslinked it with a dose of 1 J cm^− 2^. This low dose makes the coating solvent- and shear-resistant because the layer is already crosslinked, while leaving enough non-crosslinked MABP units to allow for additional photochemical reactions. For a simple initial experiment, we drop-coated a concentrated solution of the detection probes (Human IgA, 1 mg mL^− 1^) onto the mat. Then, a photomask with a 1-mm-wide transparent slit is deposited on the mat. The sample was subsequently crosslinked with a dose of 10 J cm^− 2^ and washed with water and a detergent solution (Tween) to remove the unattached protein. To test the process, we incubated the obtained test strip with a solution containing anti human IgA-Cy5 (1 µg mL^− 1^) and allowed the liquid to migrate toward the test line. After incubation, the non-specifically bound fluorescent antibodies were washed away with a Tween solution. (Fig. 8a) The resulting mat was then analyzed with a fluorescence reader yielding the fluorescence image shown in Fig. 8b. In the image, the test line is clearly visible and defined. To quantify the result, Fig. 8c. shows a plot of the intensity profile. A sharp line is clearly obtained with a signal-to-background ratio of S_b_ = 4.4. Covalent binding of the probe proteins to the detection line also ensures that no protein is washed out during migration of the sample fluid along the strip.

## Conclusions

Copolymers with photocrosslinkable groups can be used to produce fibers with a fairly homogeneous size distribution in the 800 nm range through electrospinning. The polymer molecules in the fiber can be photochemically crosslinked upon brief UV-activation due to the presence of crosslinker units. The polymers are crosslinked in the solid state within the fibers and potentially connect the fibers to each other as well. Consequently, under conditions in which non-crosslinked fibers would melt or deform, such as when they are exposed to heat or to a good solvent, crosslinked fibers exhibit no noticeable deformation of their morphology, even after prolonged exposure to adverse conditions. An advantage of the crosslinking process in the solid state is that the system consists of only one component. This process allows the use of standard polymers for electrospinning, which are mostly hydrophobic. These polymers do not swell in water and retain dimensional stability even after prolonged water immersion; however, they do not exhibit water imbibition.

The combination of fiber formation and the CHic process also allows for the simple tailoring of fiber surface chemistry and decoupling from the properties of the fiber-forming polymer. For example, thin layers of hydrophilic polymers can be readily immobilized on the fiber surface. They are not washed away when exposed to water or aqueous solutions, nor do they lose dimensional stability upon solvent exposure. Such composite materials can be produced either by dip coating the electrospun core fibers, or directly during fiber formation by coaxial electrospinning. Since the sheath around the fiber core is also crosslinked and covalently bound to the core, it is also stable against prolonged exposure to a solvent that otherwise would dissolve the coating material. This process creates a fiber network with wetting properties similar to paper, but with tailored properties. Both techniques result in a material with a contact angle against water enabling capillary flow and thus water transport across the mats.

The described composite fiber materials have the potential to substitute for paper in analytical applications requiring low background fluorescence and/or the easy attachment of biomolecules through the included crosslinker units. Further work is ongoing to improve control of the flow rates and background signals and to explore the potential for establishing new LFDs.

## Materials and methods

### Material synthesis and characterization

All raw materials and solvents were purchased from Sigma Aldrich. PMMA-co-10%-MABP and PDMAA − co-5%-MABP copolymers were synthesized by free-radical copolymerization as already described previously^22^. After degassing the reaction mixtures by three freeze and thaw cycles the polymerization was initiated using 0.1% α,α‘-azoisobutyronitrile (AIBN). The reactions were carried out at 60 °C for 15 h in DMF under nitrogen. The polymer was purified by precipitation in diethyl ether.

PMMA-co-15%-MABP: M_n_ = 230 kg mol^-1^ (GPC measurement); ^1^H NMR (250 MHz, chloroform-*d*) δ 7.88 (m, aromatic H of MABP), 7.72–7.48 (m, aromatic H of MABP), 3.66 (s, CH_3_ of MMA), 2.26–0.68 (m, polymer backbone).

PMMA-co-10%-MABP: M_n_ = 190 kg mol^-1^ (GPC measurement); ^1^H NMR (250 MHz, chloroform-*d*) δ 7.88 (m, aromatic H of MABP), 7.72–7.48 (m, aromatic H of MABP), 3.66 (s, CH_3_ of MMA), 2.26–0.68 (m, Polymer backbone).

PDMAA-co-5%-MABP: M_n_ = 190 kg mol^− 1^ (GPC measurement); ^1^H NMR (250 MHz, chloroform-d) δ 7.88 (m, aromatic H of MABP), 7.72–7.48 (m, aromatic H of MABP), 3.28–2.78 (s, CH3 of DMAA), 2.52–1.48 (m, polymer backbone).

### Viscosity measurements

For the viscosity measurements, a modular compact rheometer MCR302 from Anton Paar in conjunction with Anton Paar Rheo Compass software was used. The polymers were dissolved in DMF at various concentrations. Volumes of 70 µl were used with a 25 mm cone blade (cp25) at 30 °C. The shear rate was measured as a function of the shear rate (up to 100 Hz). The viscosity was then calculated from the slope and averaged before plotting against the concentration on a log-log scale.

### Electrospinning

**Fig. 9:**
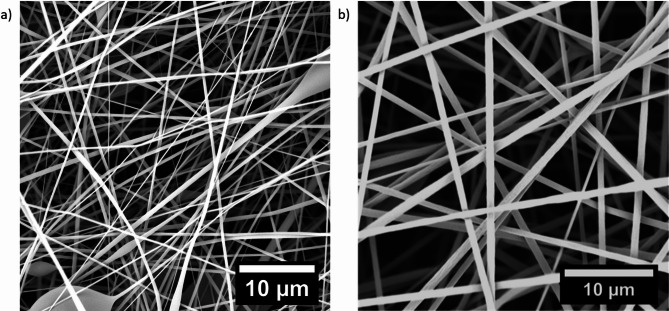
SEM pictures of the crosslinked P(DMAA-15%MABP) (a) and P(MMA-5%MABP) (b) fiber mats.

Electrospinning of the nanofiber mats was completed using the electrospinning apparatus EC-CLI with build-in HEPA filter unit produced by Innovative Mechanical Engineering Technologies B.V., Waalre, Netherlands. A 0.6 mm inner diameter (ID) capillary was used for all (non-coaxial) electrospinning experiments. The concentrations of the polymers were typically set to 20%. Temperature and humidity were adjusted based on the solvent used. For DMF, a temperature of 30 °C with a relative humidity of 20% was used. Pump speed and voltages were adjusted based on the experiment. For collection of the fibers, a drum collector wrapped in aluminum foil was used and placed at 15 cm. The lateral movement speed of the nozzle(s) was 100 mm s^− 1^ and the rotation speed of the drum was 100 rpm. Two different polymers were electrospun: P(MMA-15%MABP) and P(DMAA-5%MABP). These polymers gave nice fibers presented in Fig. 9.

For coaxial electrospinning, a coaxial nozzle supplied with the machine was used. The inner capillary, yielding the core, has a 0.6 mm ID and the outer capillary, yielding the sheath, has a 1.4 mm ID. For the PMMA-MABP (core) and PDMAA-MABP (sheath) coaxial fibers, the two materials were dissolved in DMF and pumped separately at speeds of 60 µl min^− 1^ for the both the core and sheath materials. Voltages of + 13 kV and − 3.4 kV were used for the nozzle and collector, respectively.

### Scanning electron microscopy (SEM)

The scanning electron micrographs were taken using a Phenom Pro desktop SEM (ThermoFisher Scientific). Prior to SEM imaging, the samples were sputtered with a thin layer of gold (~ 1–2 nm thick) with a Cressington Sputter Coater 108 auto from Cressington Scientific Instruments Ltd using the automatic cycle (time = 30 s, current = 40 mA). The SEM images were taken using an accelerating voltage of 10 kV in “BSD Full” mode.

### UV-crosslinking

For the electrospun fiber mats, a Bio-Link 365 UV-crosslinker with a mirrored interior from Vilber was used. The samples were placed in the middle of the chamber and irradiated for the desired input dose.

### Thermal stability

The crosslinked mats were placed in an oven pre-heated to the desired temperature (140–200 °C) for the desired time (30 min–18 h). SEM pictures were then taken from the resulting samples.

**Fig. 10:**
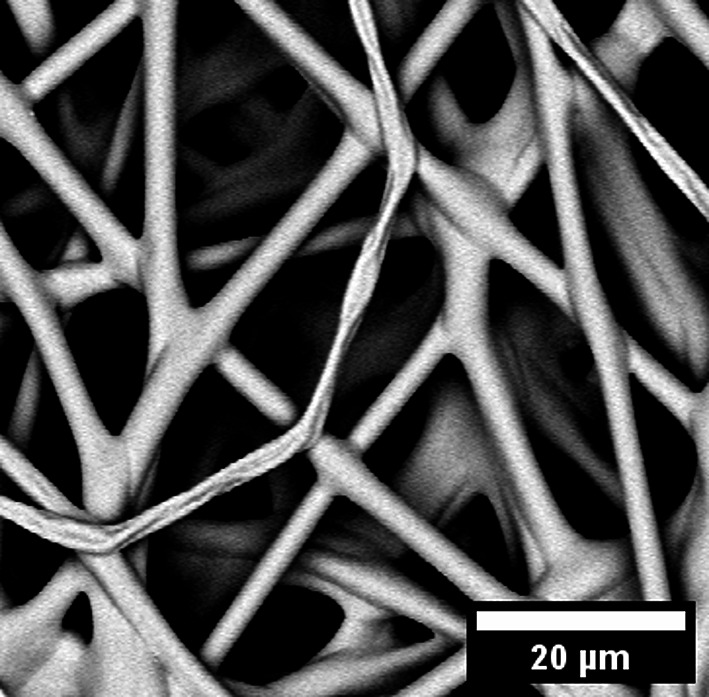
SEM picture of the crosslinked fiber mat after 18 h at 200 °C.

### Solvent stability

Solvent (toluene, acetone or ethanol) was dropped (100 µL) on the fiber mats and left to dry before taking SEM images presented in Fig. 4. In the case of longer exposure, the mat was immersed in 5 mL of Toluene for 18 h. The mat was then dried and a SEM image was taken.

**Fig. 11:**
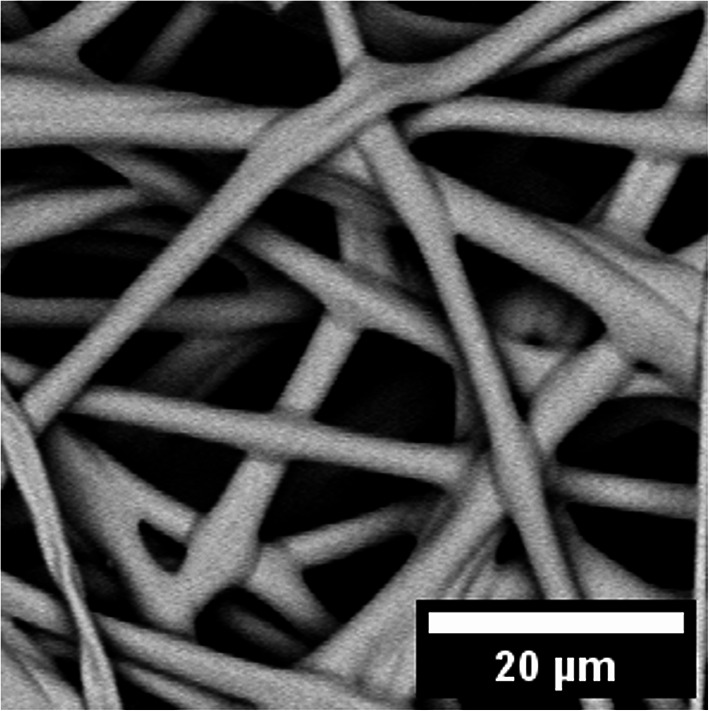
SEM picture of the crosslinked fiber mat after 18 h in Toluene.

### Dip coating

The dip coating procedures were performed using a Zwick Z 2.5 tensile testing machine manufactured by Zwick GmbH, Germany. The fiber mats were immersed in a PDMAA-5%MABP solution (1 to 10 mg.mL^− 1^ in ethanol) and draw out at a speed of 50 mm·min^− 1^.

## Data Availability

The raw datasets generated during and/or analysed during the current study are available on the FreiData system following the following link: [https://doi.org/10.60493/c5grd-93947](https:/doi.org/10.60493/c5grd-93947) .
